# Prof. Morgan’s Lectures

**Published:** 1889-05

**Authors:** John C. Morgan

**Affiliations:** Philadelphia


### PROF. MORGAN'S LECTURES.

Editors Homœopathic Physician:

I wish to be allowed a brief reply to your criticism of my lectures on *Institutes* in Hahnemann Medical College.

You are incorrectly informed as to the course; and I have to say, first, that I have only freshmen, or first young men in my class, who are unsuited to study the deep things of Hahnemann's *Organon* without a preliminary course of some extent upon the history of medicine, which the majority of the class have the good sense to perceive and profit by. If there be any individuals who fail to do so, it will surely prove a future disadvantage to them, as I think you will agree.

Secondly. Since Christmas I have lectured *only* upon the *Organon* and its teachings faithfully, and, if the earnestness of the students proves anything, most acceptably and profitably. In addition, some thirty of them have engaged in the proving of a new drug in the line of the year's work of our national society. If this be not homœopathic teaching, it seems hard to say what would be.

Very truly yours,

John C. Morgan.

Philadelphia, March 9th, 1889.

